# Xylem Sap Proteomics Reveals Distinct Differences Between *R* Gene- and Endophyte-Mediated Resistance Against Fusarium Wilt Disease in Tomato

**DOI:** 10.3389/fmicb.2018.02977

**Published:** 2018-12-04

**Authors:** Francisco J. de Lamo, Maria E. Constantin, David H. Fresno, Sjef Boeren, Martijn Rep, Frank L. W. Takken

**Affiliations:** ^1^Molecular Plant Pathology, Faculty of Science, Swammerdam Institute for Life Sciences, University of Amsterdam, Amsterdam, Netherlands; ^2^Laboratory of Biochemistry, Wageningen University, Wageningen, Netherlands

**Keywords:** endophyte, biocontrol, Fusarium wilt disease, proteomics, NP24, PR-5x, exosomes

## Abstract

Resistance (*R*) genes and endophytic organisms can both protect plants against pathogens. Although the outcome of both processes is the same, little is known about the commonalities and differences between both immune responses. Here we set out to phenotypically characterize both responses in the tomato-Fusarium pathosystem, and to identify markers to distinguish these responses at the molecular level. As endophyte *Fusarium oxysporum* (Fo) strain Fo47 was employed, which confers protection against various pathogens, including the vascular wilt fungus *F. oxysporum* f.sp. *lycopersici* (Fol). As *R*-gene conferring Fol resistance, the *I-2* gene of tomato (*Solanum lycopersicum*) was used. Fol colonizes the xylem vessels of susceptible and *I-2* resistant tomato plants, but only causes disease in the former. Fol was found to colonize the vasculature of endophyte-colonized plants, and could be isolated from stems of non-diseased plants co-inoculated with Fo47 and Fol. Because the xylem vessels form the main interface between plant and pathogen, the xylem sap proteomes during *R* gene- and Endophyte-Mediated Resistance (RMR and EMR) were compared using label-free quantitative nLC-MS/MS. Surprisingly, both proteomes were remarkably similar to the mock, revealing only one or two differentially accumulated proteins in the respective resistant interactions. Whereas in *I-2* plants the accumulation of the pathogenesis-related protein PR-5x was strongly induced by Fol, the endophyte triggered induction of both NP24, another PR-5 isoform, and of a β-glucanase in the presence of Fol. Notably, over 54% of the identified xylem sap proteins have a predicted intracellular localization, which implies that these might be present in exosomes. In conclusion, whereas both resistance mechanisms permit the pathogen to colonize the vasculature, this does not result in disease and this resistance coincides with specific induction of two distinct PR-5 isoforms and a β-glucanase.

## Introduction

Fungal plant pathogens form a major threat to food and feed production worldwide (Fisher et al., 2012). Among those, the soil-borne fungal vascular pathogen *Fusarium oxysporum* (Fo) is one of the most devastating (Dean et al., 2012). Fo encompasses more than 100 host-specific strains, so-called *formae speciales* (ff.spp.) that are typically non-pathogenic to other plant species (Gordon, 2017). Fo *forma specialis* (f.sp.) *lycopersici* (Fol) infects tomato and represents one of the best-studied Fo pathogens (Michielse and Rep, 2009). Fol does not make obvious penetration structures (e.g., appressoria), but enters the plant through natural wounds and cracks in the roots surface (Steinkellner et al., 2005). Subsequently, the fungus colonizes the apoplastic spaces of the root cortex after which it enters the stele, colonizes the xylem vasculature, and invades above-ground tissues (Olivain and Alabouvette, 1999). During infection Fol secretes a plethora of effectors to promote colonization (Houterman et al., 2007; Takken and Rep, 2010). Many of these effectors have been isolated from the xylem sap and are named Six proteins, for Secreted in xylem. One of these Six proteins is Avr2 ( = Six3), which is internalized into plant cells where it suppresses PAMP-triggered Immunity (PTI) (Di et al., 2016, 2017).

Monogenic resistance to Fol has evolved several times in tomato (Sela-Buurlage et al., 2001). A number of these *Immunity*, or *I* genes, have been cloned (Simons et al., 1998; Catanzariti et al., 2015, 2017; Gonzalez-Cendales et al., 2016) and some are bred into cultivated tomato after which their resistances have been overcome in the ongoing arms race between tomato and the fungus (Takken and Rep, 2010). The best studied *R* gene is *I-2* (van Ooijen et al., 2007), which encodes a nucleotide-binding and leucine-rich repeat (NB-LRR) protein (Simons et al., 1998) that recognizes Avr2 intracellularly to trigger *R*-gene-Mediated Resistance (RMR) (Houterman et al., 2009; Ma et al., 2013). RMR confers resistance to Fol (Houterman et al., 2009), although it permits the pathogen to colonize the vasculature to a limited extent (Mes et al., 2000; van der Does et al., 2018).

In addition to genetic resistance, endophytic microorganisms can confer protection to Fol (Fravel et al., 2003). Within the Fo species complex the vast majority of strains are harmless commensals that can colonize living plant tissues without causing disease. Fo47 represents the best-studied endophytic Fo strain. It colonizes tomato root surfaces and intercellular junctions of the root epidermis (Bolwerk et al., 2005; Olivain et al., 2006), but not the xylem vessels (Alabouvette et al., 2009). Following colonization Fo47 reduces susceptibility of the host to vascular pathogens such as Fol or *Verticillium dahliae* (Veloso and Diaz, 2012; Aime et al., 2013) thereby conferring Endophyte-Mediated Resistance (EMR).

In a compatible interaction the main interface between tomato and Fol is the xylem vessels. In agreement with this, the xylem sap proteome of susceptible plants is dramatically altered following Fol infection, and the abundance of 92% of the proteins is affected (Gawehns et al., 2015). Specifically, the abundance of stress response-related proteins is strongly increased, including Pathogenesis-Related (PR) proteins such as PR-1, PR-2 (β-glucanase), PR-3 (chitinase) and PR-5 (antimicrobial activity), and several peroxidases (Rep et al., 2002; Houterman et al., 2007; Gawehns et al., 2015). Interestingly, ≈25% of the identified xylem sap proteins does not contain a signal peptide, suggesting the non-classical secretion of the protein into the sap. Similar to tomato, a significant change in the xylem sap proteome has been observed in *Brassicca oleracea* inoculated with Fo f.sp. *conglutinans* (Foc) (Pu et al., 2016). Together, these findings show that pathogenic Fo strains affect the xylem sap proteome during infection, which might contribute to their ability to colonize the host and to cause disease.

Notwithstanding that RMR and EMR are well-studied resistance mechanisms to Fol, little is known about molecular commonalities and differences between these phenotypically indistinguishable mechanisms. Whereas it has been reported that RMR permits the pathogenic fungus to colonize the host to a limited extent (Mes et al., 2000; van der Does et al., 2018), it is currently unknown whether EMR similarly constrains the pathogen. Furthermore, it is unknown whether the composition of the xylem sap proteome is altered upon root colonization by the endophyte and/or during RMR or EMR. To obtain a better mechanistic insight in RMR and EMR – and the potential difference between these at the molecular level – the xylem sap proteomes of bi- and tri-partite interactions were determined using label-free quantitative nLC-MS/MS. The proteomes were subsequently compared with each other, and with those of mock- or Fo47-inoculated plants. In addition, the extent of host colonization by the pathogen during EMR and RMR was determined. It was found that, although the pathogen did colonize the xylem vessels, the xylem sap proteome from the disease-free EMR and RMR plants was very similar to that of non-inoculated controls. Interestingly, specific PR-5 isoforms were found to differentially accumulate during either endophyte or genetic resistance, providing excellent markers to distinguish both resistance types at the molecular level.

## Materials and Methods

### Plant and Fungal Materials and Cultivation Conditions

For fungal re-isolation assays tomato cv. KG52201 and cv. KG324 (Simons et al., 1998), respectively susceptible and resistant to Fol race 2 were used (Houterman et al., 2008). KG324 is a transgenic *I-2*-containing derivative of KG52201 (Simons et al., 1998). For plant inoculation *F. oxysporum* Fo47 (endophyte) and a Fol007 (pathogen, Fol race 2) strain carrying the *BLE* gene conferring resistance to zeocin (InvivoGen) were used (van der Does et al., 2008). For xylem sap collection the same Fol007-susceptible tomato cultivar C32 was used as before (Schmidt et al., 2013; Gawehns et al., 2015), while cv. KG324 was used as resistant cultivar. Plant inoculations where done using Fo47 (Alabouvette et al., 1987) and wild-type Fol007 (Mes et al., 1999). Plants were grown in a climate-controlled greenhouse at 24.5°C, 65% relative humidity and a 16 h photoperiod.

### Fungal Stem Reisolation Assays

Fo47 and zeocin-resistant Fol007 (FP1930) were cultured in minimal medium (0.17% Yeast Nitrogen Base without amino acids or ammonium sulfate, 3% sucrose and 100 mM KNO3) at 25°C and 150 rpm during 5 days in the dark. Cultures were filtered through Miracloth (Millipore) and diluted to yield a microconidial inoculum of 107 spores/ml (Di et al., 2016). Co-inoculum of both strains was prepared in a 1:1 ratio (10^7^ spores/ml each). For the bioassays, 10-days-old tomato seedlings were uprooted and soil was carefully removed. Roots were trimmed, leaving approximately 1 cm of roots, to facilitate fungal infection. Roots were placed for 5 min in an inoculum of Fo47, Fol007, a Fo47:Fol007 mixture, or in water without spores serving as mock control. Directly after inoculation, tomato plants were repotted and gently watered to avoid spore washing. Three weeks-post-inoculation (wpi) Fresh Weight (FW) and Disease Index (DI) were scored as described before (Gawehns et al., 2014), but adding DI = 5 when plants were dead. A statistical test (Mann–Whitney *U*) was applied on the FW and DI data using PRISM 7.0 (GraphPad). In addition, stems were harvested and surface-sterilized (Di et al., 2016). Under sterile conditions stem pieces were sectioned (0.5 cm thick approximately) at the crown- and cotyledon-level and placed on Potato Dextrose Agar (PDA) plates supplemented with 100 mg/l zeocin to specifically allow growth of Fol007. The plates contained 200 mg/l streptomycine and 100 mg/l penicillin to prevent bacterial growth. Plates were incubated in the dark at 25°C for 4 days allowing the fungus to grow out of the stem sections.

### Xylem Sap Collection

Inoculum of Fo47 and wild-type Fol007 (0.5 × 10^7^ spores/ml) and co-inoculum of both strains (0.5 × 10^7^ spores/ml each) was used to inoculate 4-weeks-old plants as described before (Schmidt et al., 2013; Gawehns et al., 2015). At two wpi, once wilt disease symptoms appeared, FW and DI were scored and xylem sap was collected from mock-treated, Fo47-, Fol007- and Fo47:Fol007-inoculated C32 plants (Bioassay 1) and mock-treated and Fol007-inoculated KG324 plants (Bioassay 2). Plants were abundantly watered 1 day and 1 h before sap collection. Tomato stems were cut below the second true leaf, and plants were placed horizontally and connected to a 12 ml- polystyrene tube placed on ice. Plants were bleeding for 6 h and the collected sap was stored at -20°C until further use (Rep et al., 2002; Krasikov et al., 2011). Bioassays were performed four times during four subsequent weeks (i.e., every single repetition equals one biological replicate per treatment). Xylem sap from 24 plants was pooled. Bioassay 1 was carried out from January to March 2017 and Bioassay 2 from October to December 2017.

### Sample Preparation for nLC-MS/MS

Potential fungal spores were removed from the sap by centrifugation at 800 ×*g* for 10 min. Xylem sap proteins were concentrated by passing 12 ml of cleared sap through Amicon Ultra-15 Filter Units (Millipore). After centrifugation at 2500 ×*g* for 15–30 min retentates containing the proteins were recovered. A BCA (bicinchoninic acid) assay (ThermoFischer) was performed to determine the protein concentration. Based on BCA quantification, a volume containing 60 μg of protein was trichloroacetic acid/aceton-precipitated and the pellet was resuspended in SDS loading buffer (2% SDS, 10% glycerol, 60 mM TRIS-HCl pH 6.8, 5% β-mercaptoethanol, 0.01% bromophenol blue), heated at 98°C for 5 min and loaded on a 12% SDS-polyacrylamide gel. Following a short electrophoresis, the proteins were stained overnight at 4°C with Commassie PageBlue (ThermoFischer). The bands containing the proteins were excised and cysteine reduction and alkylation of the proteins was performed by adding 10 mM DTT pH 8 (incubation at 60°C for 1 h) and 20 mM iodoacetamide pH 8 (incubation at room temperature in the dark for 30 min). Protein-containing gel slices were chopped into pieces of approximately 1 mm^2^ and transferred to 1.5 ml low-binding tubes (Protein LoBind microcentrifuge tubes, Eppendorf). Tryptic in-gel digestion was performed overnight by adding 50 μl of 5 ng/μl Trypsin Sequencing Grade (Sigma-Aldrich). In-house prepared μcolumns were set up by adding C18 Empore disk and LichroprepC18 column material into a 200 μl pipette tip and the tryptic peptides were eluted from the μcolumn with 50 μl of 50% acetonitrile. Acetonitrile content was reduced to <5% by reducing the volume with a concentrator at 45°C during 2 h and readjusting the volume with 1 mL/L HCOOH in water to 50 μl.

### nLC-MS/MS and Label-Free Quantification of the Proteome

Peptides were analyzed by nLC-MS/MS as previously described (Gawehns et al., 2015). Raw data from nLC-MS/MS measurements were analyzed using MaxQuant software (Cox and Mann, 2008; Hubner et al., 2010) to identify and label-free quantify the proteins. For identification of the proteins, UniProt proteome databases of tomato (UP000004994), Fo47 (UP000030766), Fol007 (UP000009097) and an in-house made contaminants database (Peng et al., 2012) were included in the Andromeda search engine (Cox et al., 2014). As before both fungal databases were improved by adding non-annotated sequences of effector proteins and predicted putative effectors (Schmidt et al., 2013). The mass spectrometry proteomics data have been deposited to the ProteomeXchange Consortium via the PRIDE (Vizcaino et al., 2016) partner repository with the dataset identifier PXD011072.

Data filtering from the MaxQuant output was carried out with Perseus 1.5.8.5. Proteins not detected by at least two peptides – of which at least one was unique and at least one was unmodified – were filtered out. Log10 transformation was applied to LFQ (Label-Free Quantification) intensities. Subsequent bioinformatics analysis was performed with R version 3.3.2.

### Gene Ontology (GO) Analysis

To sequence-annotate the identified xylem sap proteins the online webtool Mercator^1^ was used. Mercator performs (i) Blast searches against Arabidopsis TAIR 10, swiss-prot and Uniref90 databases, (ii) RPS-Blast searches against cdd and KOG and (iii) an InterPro scan. As described (Gawehns et al., 2015), specific MapMan bin-codes were assigned to each protein by Mercator and proteins were manually sorted into 10 ontology categories.

### Secretion Analysis

To determine whether the detected xylem sap proteins were secreted extracellularly, SecretomeP 2.0 and ApoplastP 1.0 web tools were used. SecretomeP (Bendtsen et al., 2004) predicts both classical protein secretion via the endoplasmic reticulum-Golgi pathway (Kehr et al., 2005) and non-classical protein secretion. SecretomeP was trained with mammalian sequences and its accuracy for plant proteomes has been questioned (Lonsdale et al., 2016), therefore ApoplastP was used as well. ApoplastP is a machine learning method that predicts apoplastic localization of proteins. ApoplastP was trained with apoplastic plant and fungal proteins (Sperschneider et al., 2018).

## Results

### Both Endophyte- and *R*-Gene-Mediated Resistance Reduce Susceptibility to Fol in Tomato Seedlings

**FIGURE 1:**
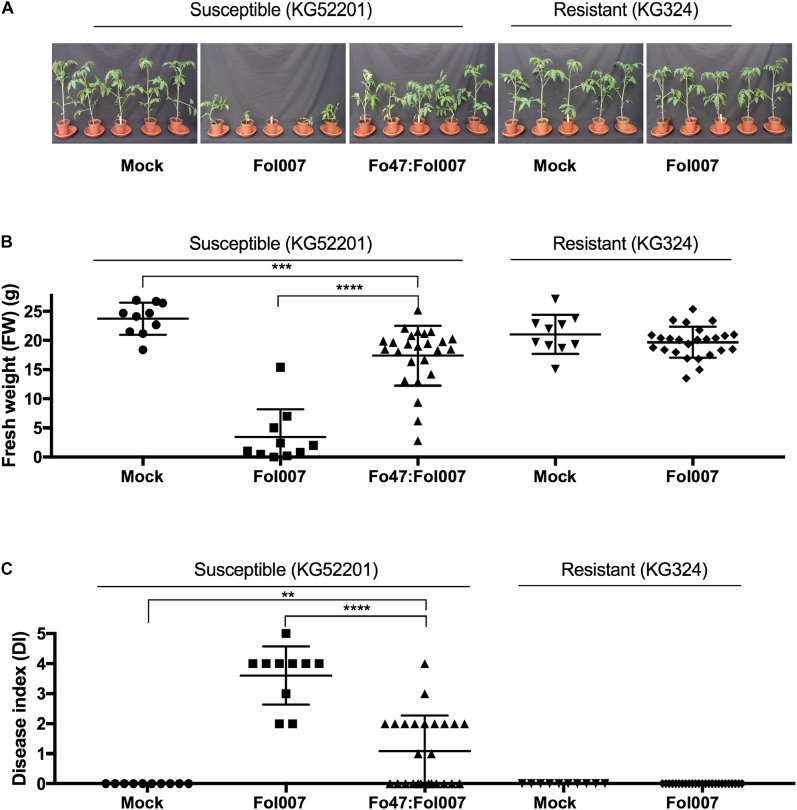
EMR and RMR reduce susceptibility to Fusarium wilt (Fol007). **(A)** Ten-days-old seedlings of Fol007-susceptible KG52201 and Fol007-resistant KG324 were root dip-inoculated with water (mock), Fol007 or a mixture of Fo47:Fol007 (25 replicates for Fo47:Fol007 co-inoculated KG52201 and Fol007-inoculated KG324 seedlings, 10 replicates for the control treatments). Three-weeks-post-inoculation **(B)** FW and **(C)** DI were scored (See Materials and Methods). The experiment was repeated three times yielding similar results (Supplementary Figures S1, S2). Error bars represent mean ±SD (^∗∗^*P_val_* < 0.01, ^∗∗∗^*P_val_* < 0.001, ^∗∗∗∗^*P_val_* < 0.0001). An unpaired comparison for FW and DI was performed using the non-parametric Mann–Whitney *U* test.

Both the fungal endophyte Fo47 (Fuchs et al., 1997) and the *I-2* resistance gene (Simons et al., 1998) have been reported to reduce susceptibility to Fusarium wilt disease in tomato. To test whether Endophyte- or *R*-gene-Mediated Resistance (EMR or RMR) also reduce susceptibility to Fol007 under our conditions, Fol007-susceptible KG52201 tomato and *I-2-*carrying resistant KG324 tomato cultivars were root dip-inoculated with either water (mock), Fol007 or a Fo47:Fol007 spore mixture (Figure 1A). Co-inoculation of KG52201 with Fo47 and Fol007 led to reduced susceptibility to Fusarium wilt disease, resulting in a significantly higher Fresh Weight (FW) than Fol007-inoculated KG52201 plants. In one repetition the FW was indistinguishable to that of the mock (Supplementary Figure S1) while in two of the three experiments, co-inoculated plants show a slightly reduced weight as compared to the mock control (Figure 1B and Supplementary Figure S2). The Disease Index (DI) (i.e., vessel browning, yellowing and wilting) was significantly reduced in co-inoculated plants as compared to Fol007-inoculated plants, but increased when compared to the mock (Figure 1C). These findings are in line with the previously reported biocontrol properties of the Fo47 strain (Fuchs et al., 1997). Inoculation of resistant *I-2*-containing KG324 plants with Fol007 revealed a strong resistance, as FW was not affected when compared to the control, and inoculated plants were disease-symptom free (DI = 0) (Figures 1B,C). Inoculation with Fo47 alone did not result in disease symptom development or a significant change in the weight of the plants (data not shown). In summary, these data show that both EMR and RMR are effective in dampening Fol007 pathogenicity resulting in a reduction, or even absence, of disease symptoms in Fol007-inoculated plants.

### Both EMR and RMR Fail in Constraining Fol Into the Root System

**FIGURE 2:**
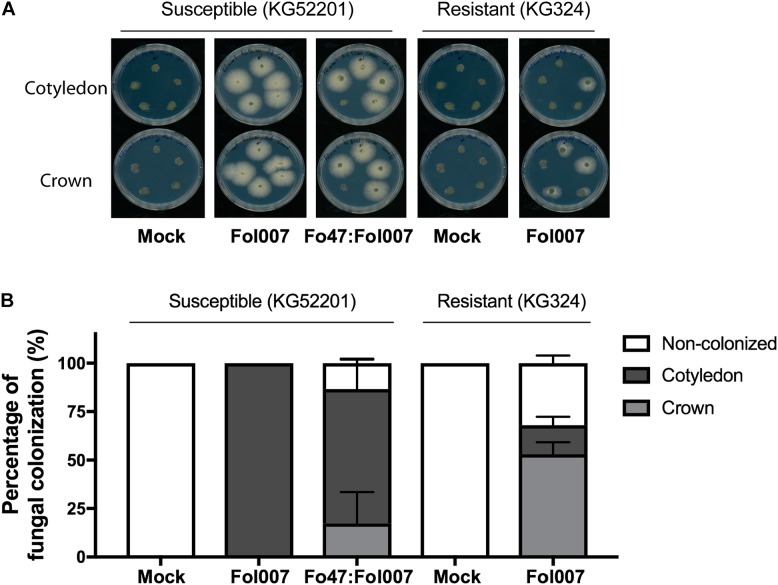
Fol007 colonizes tomato stems despite EMR or RMR. **(A)** To monitor stem colonization by Fol007 3-weeks-post-inoculation, stem sections at the crown and cotyledon-level were placed on PDA plates (25 replicates for Fo47:Fol007 co-inoculated KG52201 and Fol007-inoculated KG324 seedlings, 10 replicates for the control treatments). Plates were scanned after 4 days of incubation. **(B)** Fungal outgrowth of the stem sections plotted as a percentage of infected sections. The graph represents the combined data of three independent bioassays, error bars indicate SD.

To determine to what extent Fol007 is halted by EMR or RMR, stem sections from the above-mentioned three bioassays (Figure 1A) were collected. After surface sterilization, stem slices corresponding to the crown- and cotyledon-level were placed on PDA plates supplemented with zeocin to select for Fol007 growth, and streptomycin and penicillin to prevent bacterial growth (Figure 2A).

Stem pieces isolated from control treatments, mock-inoculated KG52201 and KG324 plants did not show any fungal outgrowth. However, fungal outgrowth was observed in all stem slices of Fol007-inoculated KG52201 plants, confirming the suitability of the technique for Fol007 re-isolation (Figure 2A). When co-inoculating susceptible KG52201 with Fo47 and Fol007 on average 17% of the plants showed Fol007 colonization only at the crown-level, whereas in 69% the fungus reached both the crown- and cotyledon-level. In 13% of the co-inoculated plants no colonization of any stem piece was observed (Figure 2B). In contrast, in Fol007-inoculated KG324 plants the fungus was halted at the crown-level in 53% of the replicates and only in 15% reached also the cotyledon-level, while in 32% no colonization was found. We conclude that RMR (i.e., genetic resistance) is more effective than EMR in restricting fungal growth.

### EMR and RMR Reduce Susceptibility to Fol in 4-Weeks-Old Tomato Plants

**FIGURE 3:**
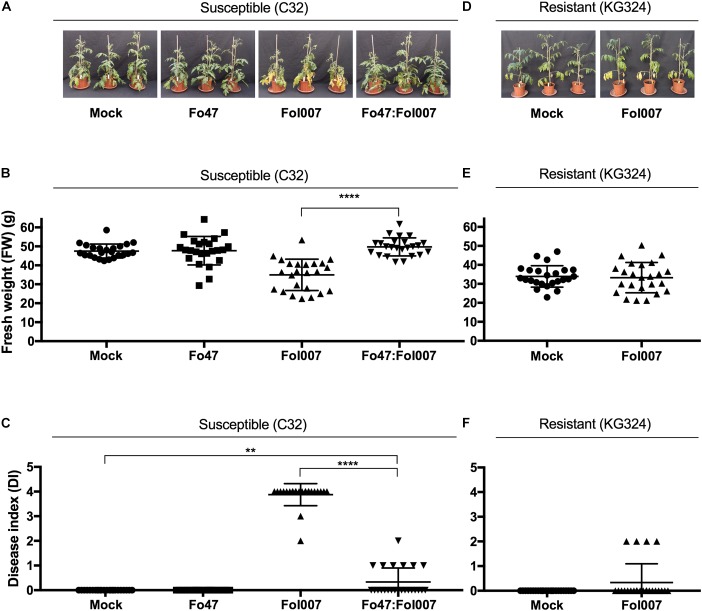
Disease symptoms induced by Fol007 are reduced by EMR or RMR in 4-weeks-old plants. **(A)** 4-weeks-old Fol007-susceptible KG52201 plants were inoculated with water (mock), Fo47, Fol007 or a mixture of Fo47:Fol007 (24 plants/replicate). Two-week-post inoculation **(B)** FW and **(C)** DI were scored (See Materials and Methods). In an independent experiment **(D)** 4-weeks-old Fol007-resistant KG324 plants were inoculated with water (mock) or Fol007. Two-week-post inoculation **(E)** FW and **(F)** DI were scored. **(A–C)** Correspond to the Bioassay 1 and **(D–F)** to Bioassay 2. Error bars represent mean ±SD (^∗∗^*P_val_* < 0.01, ^∗∗∗∗^*P_val_* < 0.0001). An unpaired comparison for FW and DI was performed using the non-parametric Mann–Whitney *U* test.

Next, we tested whether EMR and RMR also reduce susceptibility to Fol007 infection of 4-weeks-old plants that can be used for xylem sap isolation. Four-weeks-old Fol007-susceptible C32 plants were root dip-inoculated with water (mock), Fo47, Fol007 or a mixture of Fo47:Fol007 (Figure 3A). This bioassay was repeated four times. Two weeks-post-inoculation (wpi), FW of Fo47:Fol007 co-inoculated C32 plants was higher than that of Fol007-inoculated C32 plants and indistinguishable from the mock in two of the four bioassays (Figure 3B). In the other two assays, no significant differences in FW were found. Regarding the DI, Fo47:Fol007 co-inoculated C32 plants showed a strongly reduced DI as compared to Fol007-inoculated plants, but an increased DI when compared to the mock (Figure 1C). This pattern was consistent in all four repetitions. Hence, EMR is effective in 4-weeks-old tomato plants and results in a strong reduction of the DI as compared to the susceptible control and in no discernible difference in weight in two of the four assays as compared to the mock.

Concerning resistant KG324 plants, 4-weeks-old plants were root dip-inoculated with water (mock) or Fol007 (Figure 3D). Scoring the plants two wpi revealed that FW and DI where not significantly altered following Fol007-inoculation as compared to the mock (Figures 3E,F). This pattern was consistently observed in four repetitions showing the strong protection conferred by RMR to Fol007. In brief, both EMR and RMR are effective in reducing Fusarium wilt disease in 4-weeks-old plants.

### Fo47 Does Not Affect the Tomato Xylem Sap Proteome in Bi-partite Interactions

Infection of susceptible tomato with pathogenic Fol007 strongly affects the tomato xylem sap proteome (Gawehns et al., 2015). To investigate whether Fo47 inoculation also affects the proteome, and whether putative changes correlate with biocontrol abilities of the fungus, nLC-MS/MS analysis was performed on xylem sap collected from Fo47-inoculated tomato plants. The relative abundance of the identified protein was then compared to that of mock-inoculated plants.

**FIGURE 4:**
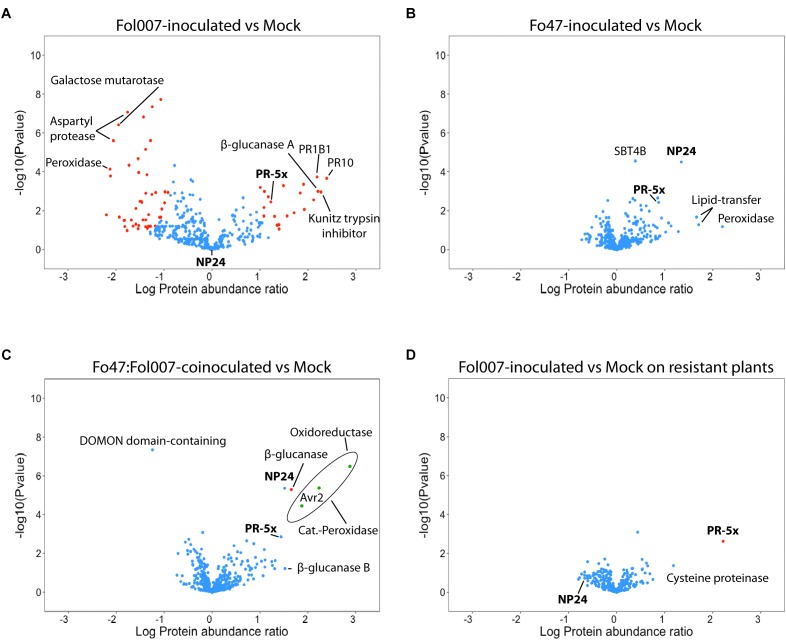
Bi- and tri-partite interactions between tomato, Fo47 and Fol007 differentially affect the xylem sap proteome. Volcano plots showing the comparison between –log10 Pvalue (*y*-axis) and log10 Fold change (*x*-axis) of two different treatments (four biological replicates each). Blue dots represent proteins whose abundance did not change among the compared treatments, whereas red dots are DAPs. Green dots represent fungal DAPs. PR-5 isoforms are marked in bold font and the others correspond to the top up- or down-accumulated proteins (up to eight). **(A)** Xylem sap proteome of susceptible C32 plants inoculated with Fol007, **(B)** Fo47 and **(C)** Fo47*:*Fol007-coinoculated as compared to mock-inoculated C32. Also, **(D)** xylem sap proteome of resistant KG324 plants inoculated with Fol007 as compared to mock-inoculated KG324. PR1B1 on 4A panel refers to Pathogenesis-related leaf protein 6.

As reported before, Fol007 infection causes a major shift in the relative abundance of many xylem sap proteins as compared to the mock (Gawehns et al., 2015). However, fewer Differentially Accumulated Proteins (DAPs) were found in the current study due to a more stringent cutoff as a S0 index was used, which besides the *P*-value also takes fold-change into account (Figure 4A). Out of the 388 proteins found in xylem sap of mock- and Fol007-infected tomato plants, 71 were DAPs (Supplementary Table S1). The protein with the highest fold-change was PR-10 (K4CWC5), which accumulated 246-fold. Six β-glucanases were identified showing distinct accumulation patterns: β-glucanases Q01412 and K4BBH7 accumulated 161-fold and 31-fold respectively, whereas quantities of the other four decreased (K4BZT8 158-fold, K4B3H0 66-fold, K4CF40 24-fold, and K4CMF9 10-fold). A similar pattern was observed for chitinases. Two basic chitinases (K4B667 and P32045) accumulated 131-fold and 84-fold while the quantity of an acidic chitinase (Q05540) decreased 59-fold. Notably, in the xylem sap of Fo47-inoculated plants no DAPs were identified among the 388 proteins, implying that colonization by the endophyte (Supplementary Figure S4) does not affect the xylem sap proteome. Furthermore, no Fo47-specific proteins were detected in the sap (Figure 4B).

### EMR Significantly Affects the Abundance of a Single Tomato Xylem Sap Protein

Fo47 does not affect the xylem sap proteome of tomato in a bi-partite interaction (Figure 4B). However, it is not known whether Fo47 is able to do so when EMR is triggered by co-inoculation with Fol007 (Figure 3) and the pathogen colonizes the shoot (Figure 2). To examine this, xylem sap protein abundance of Fo47:Fol007-coinoculated C32 plants was compared to mock-inoculated plants (Figure 4C) and, surprisingly, only a single DAP was identified. This protein, a β-glucanase (K4BBH7), was detected by 11 unique peptides and its abundance was increased 45-fold as compared to the mock. Besides this protein also NP24 – a PR-5 isoform – highly accumulated (33-fold), but this protein was not identified as a DAP due to the stringent S0 settings applied. Hence, even though Fol007 dramatically affects the tomato xylem sap proteome in bi-partite interactions, the proteome remained almost unaltered when the pathogen is co-inoculated with Fo47.

When Fol007 was inoculated alone, 39 fungal proteins were found of which 36 were identified as DAPs according to their LFQ intensity values (Supplementary Table S2). Upon co-inoculation 13 fungal proteins were identified in the xylem sap of which three were DAPs (Figure 4C, green dots). These three proteins were, from lowest to highest abundance, the Fol007 effector Avr2 (A0A0C4DI32), a catalase-peroxidase (A0A0J9W9G5) and an oxidoreductase (A0A0C4DHX8). The LFQ values show a reduced accumulation of these Fol007 proteins upon co-inoculation with Fo47.

Notably, when Fo47 was inoculated alone or co-inoculated with Fol007 nine tomato xylem sap proteins were specifically induced and these were not detected following mock- or Fol007-inoculation (Supplementary Figure S3A). These proteins were present in relatively low quantities therefore they were not assigned as DAPs (Supplementary Table S1). These proteins represent a β-glucosidase 08 (B5M9E5), Aspartyl protease (K4AXP3), GDSL-like Lipase/Acylhydrolase (K4B1C4), Cysteine proteinase (K4B7P1), Unknown protein (K4BJU1), Zn-dependent exopeptidase (K4BJY1), Aspartyl protease (K4C3U1), aldehyde dehydrogenase (K4C8H3) and a Glycosyltransferase (K4D3D1).

Next, a comparison was made between the xylem sap proteomes of Fo47:Fol007-coinoculated plants versus Fo47-inoculated C32 to identify DAPs that are potentially linked to reduced susceptibility to Fol007 (Supplementary Figure S3B). Within this set of 393 proteins eight were identified as DAPs. These are two glucanases (K4BBH7, Q01412), two peroxidases (K4ASJ7, K4C1C1), a Aconitate hydratase (K4CFD4), a FASCICLIN-like arabinogalactan protein (K4C9N8) and a Lipid transfer protein (K4B273). Notably also a Leucine-rich repeat protein kinase (K4BK30) was found, which is typically a plasma membrane localized protein (Supplementary Table S5).

Taken together, inoculation with the endophyte alone or in combination with Fol007 pathogen, results in highly similar proteome profiles as those of the mock. Comparison of Fo47:Fol007 with the mock results in a single DAP while comparison to Fo47-inoculated plants reveals eight DAPs.

### RMR Significantly Affects the Abundance of a Distinct PR-5 Isoform

To identify proteins indicative for RMR, the xylem sap proteomes of mock- and Fol007-inoculated *I-2-*containing tomato plants were compared. As observed for EMR, the proteome profiles of mock and Fol007-inoculated are highly similar showing only a single DAP. Accumulation of this DAP, PR-5x (Q8LPU1), was strongly induced (158-fold) following pathogen inoculation as compared to the mock (Figure 4D).

Identification of two PR-5 family members being strongly induced in either RMR (PR-5x) or EMR (NP24) is intriguing, as it implies an important role for this class of proteins in resistance to Fusarium wilt in tomato. Within the xylem sap proteomes 12 PR-5 proteins were identified: PR-5x (Q8LPU1), NP24 (P12670), five osmotin-like proteins (K4CP63, Q01591, K4CP65, K4CP64, K4CP59) and five thaumatin-like proteins (K4BV68, K4BVN4, K4BAP4, K4DFX0, K4BBQ9) (Supplementary Table S4).

In the xylem sap of a compatible interaction between susceptible C32 tomato and Fol007, a 17-fold increase in PR-5x accumulation was observed as compared to the mock (Figure 4A). Notably, in these plants accumulation of NP24 is not altered and the absolute quantity of this protein is lower than that of PR-5x. In contrast, when tomato is inoculated with Fo47 NP24 accumulated 22-fold as compared to the mock while PR-5x is only induced to a limited extent (7-fold change vs. mock) (Figure 4B). Upon co-inoculation of tomato with Fo47 and Fol007 both PR-5 isoforms are induced to a similar level: PR-5x accumulates 28-fold and NP-24 33-fold compared to the mock control (Figure 4C).

Altogether, these results show that PR-5x accumulation is specifically induced upon pathogen infection in both compatible and incompatible interactions of tomato with Fol007. But although induced in both cases, the induction of PR-5x in RMR is much higher than that in a diseased susceptible plant (158- vs. 17-fold). In Fo47 inoculated plants NP24 accumulation was induced 22-fold in the absence-, and 33-fold in the presence of Fol007. The remarkable difference between the relative accumulation between these two PR-5 isoforms in RMR and EMR indicate that they can be used as markers to distinguish both resistances types on the molecular level.

### Many Xylem Sap Proteins Have a Predicted Intracellular Localization

It has been reported that ≈75% of the tomato xylem sap proteins carry a signal peptide for extracellular secretion (Gawehns et al., 2015). Of the remaining proteins, ≈10% was predicted to be non-classically secreted (Gawehns et al., 2015) whereas the other ≈15% has a proposed intracellular localization. Here we set out to investigate whether similar ratios of putative extracellular- and cytoplasmic-localized proteins are present in our current datasets (Supplementary Tables S1, S3). The percentage of predicted extracellular and intracellular proteins in the xylem sap proteins of non-inoculated and Fol007-inoculated susceptible plants was analyzed using two different algorithms: SecretomeP (Bendtsen et al., 2004) and ApoplastP (Sperschneider et al., 2018).

**FIGURE 5:**
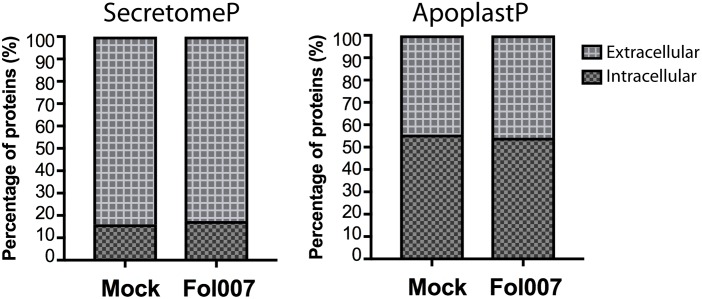
The xylem sap proteome contains predicted intracellular proteins. Bar plots representing the percentage of intracellular or extracellular tomato xylem sap proteins found in mock- and Fol007-inoculated plants. Prediction was carried out by the webtools SecretomeP **(left)** and ApoplastP **(right)**.

As reported SecretomeP predicted the majority of the proteins to be secreted to extracellular spaces (Gawehns et al., 2015). For both mock- and Fol007-inoculated plants 74% of all proteins identified are predicted as conventionally secreted proteins, 9% as leaderless secreted proteins and 17% as being non-secreted (Figure 5). ApoplastP predicted respectively 44 and 46% of the proteins from mock- and Fol007-inoculate to be secreted. Hence, the majority of the xylem sap proteins (>54%) is not identified by the program as a putative extracellular protein. The difference between both programs is remarkable, but nevertheless both predict a large subset of proteins to be localized intracellularly. In conclusion, both SecretomeP and ApoplastP predict an intracellular location for a large population of the xylem sap proteins.

## Discussion

Here, we show that Endophyte-Mediated Resistance (EMR) and *R-*gene-Mediated Resistance (RMR) have remarkably similar xylem sap proteome profiles. Upon Fol007 inoculation of *I-2-*containing resistant KG324 tomato plants (i.e., RMR) the accumulation of the vast majority of xylem sap proteins was similar to that of the mock. Strikingly, out of 303 tomato proteins identified, PR-5x was the only DAP and its abundance was increased by 158-fold. Contrarily, a different PR-5 isoform (NP24) specifically accumulated upon (co)inoculation of Fo47 on susceptible C32 tomato (i.e., EMR). Upon Fo47 and Fol007 co-inoculation also a β-glucanase, forming the only DAP in this dataset, was specifically induced. Within this tri-partite interaction between Fol007, Fo47 and C32 tomato, several Fol007 proteins were found in the xylem sap showing that EMR does not restrict the ability of Fol007 to colonize the xylem vessels. Furthermore, the major changes in the proteome profile that are observed in susceptible interactions, and are concomitant with disease symptoms, are not observed during EMR.

In compatible interactions with susceptible tomato plants Fol007 dramatically alters the xylem sap proteome (Figure 4A). Contrarily, Fo47 inoculation alone did not significantly affect the xylem sap proteome. As shown in Supplementary Figure S4 the fungus could be re-isolated from stems of Fo47-inoculated tomato plants. However, not a single Fo47 protein was found in the xylem sap from either Fo47-inoculated or Fo47:Fol007-coinoculated tomato plants, which indicates that the endophyte does not colonize the vasculature or at least not in detectable amounts. This conclusion is in correspondence with previous reports describing Fo47 as a colonizer of root surfaces and of intercellular junctions of the root epidermis (Bolwerk et al., 2005; Olivain et al., 2006) and not being able to reach the xylem vessels (Alabouvette et al., 2009). Nine tomato proteins were exclusively detected in low quantities upon inoculation with Fo47 regardless of the presence of Fol007. The specific accumulation of these proteins might be linked to the colonization and concomitant biocontrol conferred by the endophyte (Supplementary Figure S3A and Supplementary Table S1). The genes encoding these proteins could be targeted in future study to assess their potential involvement in these processes.

When Fo47 is co-inoculated with Fol007 the latter retained its ability to colonize the xylem vessel (Figure 2 and Supplementary Table S2). The identification of 13 Fol007 proteins in the sap upon co-inoculation with Fo47 confirms the presence of Fol007 in the xylem. The number and abundance of identified Fol007 proteins in EMR is, however, lower than that in susceptible plants. Together with a reduced DI, this suggests that Fol007 biomass is reduced by EMR. Since both fungi appear not to co-exist in the xylem vessels, this implies that EMR is mediated by the plant. This idea is supported by the finding that Fo47 induces resistance to Fol in a tomato split-root system (Fuchs et al., 1997). Moreover, it has been demonstrated that Fo47 can prime defense responses and reduce susceptibility to wilt disease when inoculated before Fol inoculation (Aime et al., 2013). Since it takes Fol007 at least 2 days to reach the vasculature (van der Does et al., 2018), it appears that defense priming induced by Fo47 suffices to limit the ability of the pathogen to extensively colonize the vessels and cause disease. The xylem sap proteins involved in this process are unknown, but the eight DAPs obtained in the proteome comparison between Fo47:Fol007-co-inoculated and Fo47-inoculated susceptible plants represent good candidates to be involved in this process (Supplementary Table S5). In future studies, the genes for these proteins could be targeted to assess their potential involvement in EMR.

When resistant *I-2*-containing tomato plants where inoculated with Fol007, not a single fungal protein was identified, although the fungus could be isolated from the infected stem pieces (Figure 2). A similar strong reduction in the ability of Fo to colonize the vessels of a resistant host was observed in *B. oleracea* cultivars challenged with Foc, and Foc fungal proteins were only detected in xylem sap of susceptible plants (Pu et al., 2016). These observations suggest that the amount of fungal biomass in the vessels is strongly reduced by RMR, which is in agreement with the complete absence of disease symptoms. The absence of symptoms and the presence of the fungus in the vessels led to the proposition that *R* genes switch the lifestyle of a pathogenic *Fusarium* into that of an endophyte (van der Does et al., 2018). Our data support this hypothesis, by showing that not only disease symptoms are absent, but also the proteome is essentially identical to that of non-inoculated- or endophyte-inoculated plants.

β-glucanases are among the most commonly detected proteins within the tomato xylem sap. In fact, 17 β-glucanases were detected in Bioassay 1 and 2, but only a single β-glucanase (K4BBH7) was identified as a DAP upon co-inoculation of Fo47 and Fol007 (Supplementary Tables S1, S3). These enzymes target β-glucans, which present the most abundant fungal cell wall polysaccharides. Together with chitin glucans form the main structural component of the cell walls. Enzymatic attack of this network by β-glucanases has a direct antimicrobial effect by perturbation of the fungal cell wall integrity (Stintzi et al., 1993). In addition, the subsequent release of β-1,6-glucans, which are specific to fungi and members of the Stramenopiles, can elicit PTI in many plant species (Fesel and Zuccaro, 2016). To avoid glucan degradation and subsequent PTI activation the fungal endophyte *Serendipita indica* (formerly *Piriformospora indica*) secretes a fungal-specific lectin (FGB1). FGB1 binds β-glucan-and suppresses glucan triggered immunity, thereby facilitating fungal host colonization (Wawra et al., 2016). Whether Fo47 or Fol007 produce secreted lectins is currently unknown. Fungal lectins have not been identified in the xylem sap of infected plants in the current study nor in a former analysis, although LysM-containing proteins have been identified in this former study and these are proposed to sequester released chitin (Gawehns et al., 2015). Whether the identified β-glucanases play a role in biocontrol and/or restriction of disease symptom development during EMR remains to be tested.

Endophytic interactions of Fo47 with tomato resulted in a 22-fold induction of the PR-5 isoform NP24 (Figure 4B). This protein accumulated 33-fold higher in a tri-partite interaction with Fol007 (Figure 4C), but was not identified as a DAP due to stringent setting of the S0 index. However, when the same settings are applied as used previously (Gawehns et al., 2015) NP24 is identified as a significant DAP and the protein is no longer identified in the mock. In addition, the amount of NP24 might be underestimated in our setup. A limitation of using LFQ values is that identification of a protein relies on the presence of at least two tryptic peptides, of which at least one is unique. When proteins belong to a family sharing high sequence similarity, a non-unique peptide is assigned only to a single member of the family, resulting in the underestimation of the amounts of the other members. For instance, NP24 is detected by five peptides of which three are unique but two are non-unique and are assigned to PR-5x (Supplementary Table S6). When only the unique peptides are used for protein quantification, then its absolute amount is underestimated (i.e., LFQ values will be reduced) but the calculated fold change is more precise. When quantification of NP24 is done based on unique peptides only, then the accumulation in the Fo47-inoculated plants differs significantly from the mock as the protein is absent in the latter (Supplementary Table S7). NP24 has *in vitro* antimicrobial activity against *Phytophthora infestans* (Woloshuk et al., 1991) and is involved in resistance of ripe pepper fruits to *Colletotrichum gloeosporioides* (King et al., 1988; Oh et al., 2003). The correlation between NP24 protein abundance and reduced disease symptoms during EMR could indicate that the protein might also have antifungal activity *in planta*, thereby reducing the extent of Fol007 colonization of the vasculature.

PR-5x accumulation was specifically induced in the xylem sap of resistant (158-fold) and susceptible (17-fold) tomato plants upon Fol007 infection (Figure 4A). PR-5x was identified previously in tomato xylem sap following Fol infection of susceptible and resistant plants, hence its name PR-5xylem (Rep et al., 2002). The protein is distinct from the PR-5 isoforms present in apoplastic fluid of tomato leafs infected by *Cladosporium fulvum* and it is not present in the latter (Joosten and De Wit, 1989; Rep et al., 2002). Compared to other PR-5 isoforms, the gene encoding PR-5x shows a root-specific expression (Rep et al., 2002). A similar xylem sap-specific accumulation of PR-5 isoforms has been observed upon Foc infection of Foc-resistant and -susceptible cultivars of *B. oleracea* (Pu et al., 2016). Colonization of *Arabidopsis thaliana* roots by non-pathogenic fluorescent *Pseudomonas* spp. triggers induction of *AtTLP1*, a PR-5 family member, in the vascular bundle, which is related to a local response to colonization of this non-pathogenic bacterium (Leon-Kloosterziel et al., 2005). It has also been reported that accumulation of PR-5 isoforms in the xylem sap of tomato is strongly induced in plants inoculated with Fol strains in which key effectors, such as Avr2, Six1 and Six6 have been knocked out. These Fol mutants were still capable of causing disease, yet the amount of fungal biomass in the plant was strongly reduced concomitant with a reduction in disease symptoms. Specifically, NP24 accumulated > 200-fold in plants inoculated with a *SIX1* or *SIX6* knockout Fol strain in comparison to inoculation with wild type Fol. Similarly, a Thaumatin (K4DFX0) accumulated strongly when Avr2 was knocked out (Gawehns et al., 2015). Based on these observations it is tempting to speculate that antimicrobial activity of PR-5 isoforms might be the main determinant controlling the amount of *Fusarium* biomass in the xylem vessels. Antimicrobial activity of these small ± 24 kDa cysteine-rich proteins has been described before (Hoffmann-Sommergruber, 2000; Chowdhury et al., 2015). PR-5 proteins disrupt the lipid bi-layer of fungi, resulting in the formation of transmembrane pores that cause a strong permeability of the plasma membrane (Vigers et al., 1992). In addition to its antimicrobial activity, PR-5 proteins may themselves trigger plant defense responses. Transgenic Arabidopsis plants over-expressing *Prunus domestica PR5-1* show induction of the *phenylalanine ammonia-lyase (PAL)* gene and a concomitant increased flux through the phenylpropanoid biosynthesis pathway. Moreover, genes involved in the biosynthesis of camalexin (a phytoalexin), which is an endproduct of the phenylpropanoid pathway, are up-regulated in these transgenic plants. *PdPR5-1* Arabidopsis plants also showed an increased resistance to *Alternaria brassicicola* (El-Kereamy et al., 2011). It is imaginable that that PR-5x and NP24 have a function similar as *PdPR5-1* and confer both direct and indirect resistance to *F. oxysporum* in tomato. The identification of two distinct PR-5 family members, whose induced expression correlates with either RMR and EMR, shows that both resistance mechanisms independently induce the expression of a specific PR-5 isoform to control pathogen proliferation in the plant. The underlying mechanism remains to be elucidated, but the PR-5 isoforms identified provide excellent markers to distinguish both immune responses.

ApoplastP and SecretomeP predicted many of the proteins detected in the xylem sap to be localized intracellularly (Figure 5 and Supplementary Tables S1, S3). It is intriguing that predicted cytosolic proteins appear in the xylem sap, which is an extracellular space. A possible explanation is that cellular damage caused by Fol007 releases proteins from xylem adjacent cells. However, these proteins were also present in the mock treatments making this explanation unlikely. In addition, only a relatively small number of intracellular proteins in processes like photosynthesis (2% of all identified proteins) or primary metabolism (2%), or TCA cycle (1%) were found, which is suggestive for a specific secretion mechanism rather than generic loss of cellular integrity. A recently proposed mechanism for this is that these putative ‘intracellular’ proteins are contained in exosomes. Since long it is known that plant cells can secrete exosomes into extracellular spaces (Halperin and Jensen, 1967), but little was known about their role in plant-microbe interactions. Recently, exosomes were isolated from the apoplastic fluid of *A. thaliana* and these were found to be enriched in proteins related to stress and plant defense (Rutter and Innes, 2017). In fact, homologues proteins identified in that study were also present in our datasets. Among these are Leucine-rich repeat receptor-like protein kinase (K4D401), Early nodulin-like protein (K4CXN7), Lipase/Lipooxygenase and PLAT/LH2 family protein (K4BIL3). Together with our observation that three proteins involved in vesicle-mediated trafficking (K4B1S4, K4CPC9 and K4D8S6) were identified in the tomato xylem sap, this could imply that exosomes may play a role in the interaction between Fo and tomato.

In this study two different PR-5 isoforms were identified whose abundance correlate with a reduction in pathogen biomass. Furthermore, these PR-5 proteins serve as specific markers for either RMR (PR-5x) or EMR (NP24). Current studies, in which these genes are knocked out or overexpressed in tomato, could address the question whether their specific increase in abundance is causal to Fol007 resistance. It will be interesting to investigate accumulation of extracellular PR-5 members in other Fo-plant pathosystems, such as melon containing the *Fom2* resistance gene and *F. oxysporum f. sp. melonis*, to elucidate whether increased accumulation of PR-5 members is a conserved feature of resistance to Fusarium wilt disease. If so, this finding will yield exciting possibilities for novel strategies for resistance breeding in crop species where dominant resistance genes are unavailable, such as banana, or when use of endophytes is not applicable or feasible.

## Author Contributions

FT and FL designed the experiments. FL and MC performed the fungal stem re-isolation assays. FL, MC, and DF collected the xylem sap. FL prepared the samples for nLC-MS/MS measurements. SB performed the nLC-MS/MS measurements. FL and SB analyzed the data. MR gave intellectual input and critically revised the manuscript. FL and FT wrote the manuscript.

## Conflict of Interest Statement

The authors declare that the research was conducted in the absence of any commercial or financial relationships that could be construed as a potential conflict of interest.

## Footnotes

1 http://mapman.gabipd.org

## Supplementary Material

FIGURE S1 EMR and RMR reduce susceptibility to Fusarium wilt (Fol007). **(A)** Ten-days-old seedlings of Fol007-susceptible KG52201 and Fol007-resistant KG324 were root dip-inoculated with water (mock), Fol007 or a mixture of Fo47:Fol007 (25 replicates for Fo47:Fol007 co-inoculated KG52201 and Fol007-inoculated KG324 seedlings, 10 replicates for the control treatments). Three-weeks-post-inoculation **(B)** FW and **(C)** DI were scored (See Materials and Methods). The experiment was repeated three times yielding similar results (Figure 1 and Supplementary Figure S2). Error bars represent mean ±SD (^∗∗^*P_val_* < 0.01, ^∗∗∗^*P_val_* < 0.001, ^∗∗∗∗^*P_val_* < 0.0001). An unpaired comparison for FW and DI was performed using the non-parametric Mann–Whitney *U* test.

FIGURE S2 EMR and RMR reduce susceptibility to Fusarium wilt (Fol007). **(A)** Ten-days-old seedlings of Fol007-susceptible KG52201 and Fol007-resistant KG324 were root dip-inoculated with water (mock), Fol007 or a mixture of Fo47:Fol007 (25 replicates for Fo47:Fol007 co-inoculated KG52201 and Fol007-inoculated KG324 seedlings, 10 replicates for the control treatments). Three-weeks-post-inoculation **(B)** FW and **(C)** DI were scored (See Materials and Methods). The experiment was repeated three times yielding similar results (Figure 1 and Supplementary Figure S1). Error bars represent mean ±SD (^∗∗^*P_val_* < 0.01, ^∗∗∗^*P_val_* < 0.001, ^∗∗∗∗^*P_val_* < 0.0001). An unpaired comparison for FW and DI was performed using the non-parametric Mann–Whitney *U* test.

FIGURE S3 Fo47 inoculation affects accumulation of specific proteins. **(A)** Proteins that were detected in at least three out of four replicates where labeled as present. Treatments embraced mock-inoculated, Fo47-inoculated, Fo47:Fol007-coinoculated and Fol007-inoculated C32 susceptible tomato plants. The black circle marks proteins that were specifically present in Fo47 inoculated plants. **(B)** Volcano plot showing xylem sap protein abundance difference between Fo47:Fol007-coinoculated compared to Fo47-inoculated on susceptible C32 plants. Names of the DAPs are depicted in Supplementary Table S5.

FIGURE S4 Fo47 colonizes tomato stems **(A)** To monitor stem colonization by Fo47 3-weeks-post-inoculation, stem sections at the crown and cotyledon-level were placed on PDA plates (10 replicates). Plates were scanned after 4 days of incubation. **(B)** Fungal outgrowth of the stem sections plotted as a percentage of infected sections. The experiment was repeated three times showing the same pattern.

TABLE S1 Tomato proteins identified and label-free quantified in Bioassay 1. Samples labeled as ‘Mock’ for the water control, and ‘Endo,’ ‘Coinoc’ and ‘Patho’ for Fo47-inoculated, Fo47:Fol007-coinoculated and Fol007-inoculated C32 susceptible tomato plants. DAPs are marked with +. LFQ values are represented in Log10 scale. Sequence coverage (%), molecular weight (KDa), GO description and SecretomeP and ApoplastP outputs are provided for all proteins.

TABLE S2 Fol007 proteins identified and label-free quantified in Bioassay 1. LFQ values are in Log10 scale and 3,9 represent the LFQ cutoff.

TABLE S3 Tomato proteins identified and label-free quantified in Bioassay 2. Samples labeled as ‘Mock’ for the water control, and ‘Patho’ for Fol007-inoculated KG324 resistant tomato plants. DAPs are marked with +. LFQ values are represented in Log10 scale. Sequence coverage (%), molecular weight (KDa), GO description and SecretomeP and ApoplastP outputs are provided for all the proteins.

TABLE S4 List of PR-5 proteins found in xylem sap of both bioassays. Uniprot IDs, names of the proteins, average of LFQ intensities for every treatment of the two bioassays, predicted location, GO category, disulphide bonds and length of the proteins are depicted in the table. LFQ values are represented in Log10 scale.

TABLE S5 List of xylem sap proteins whose relative abundance changes upon co-inoculation of tomato with Fo47:Fol007 comparing to Fo47-inoculated.

TABLE S6 Overview of the peptides matching NP24 and PR-5x proteins.

TABLE S7 Overview of the protein groups detected peptides overlapping with NP24 and PR-5x. Filled in green the LFQ intensities obtained by only using unique peptides for the protein quantification. LFQ intensities are represented in Log10 scale.
