# Corrigendum: Xylem Sap Proteomics Reveals Distinct Differences Between *R* Gene- and Endophyte-Mediated Resistance Against Fusarium Wilt Disease in Tomato

**DOI:** 10.3389/fmicb.2019.01872

**Published:** 2019-08-13

**Authors:** Francisco J. de Lamo, Maria E. Constantin, David H. Fresno, Sjef Boeren, Martijn Rep, Frank L. W. Takken

**Affiliations:** ^1^Molecular Plant Pathology, Faculty of Science, Swammerdam Institute for Life Sciences, University of Amsterdam, Amsterdam, Netherlands; ^2^Laboratory of Biochemistry, Wageningen University, Wageningen, Netherlands

**Keywords:** endophyte, biocontrol, Fusarium wilt disease, proteomics, NP24, PR-5x, exosomes

In the original article, there was an error. The amount of xylem sap protein used for nLC-MS/MS analysis was incorrectly depicted; instead of 540 μg of protein 60 μg of protein was TCA precipitated and used for SDS-polyacrylamide gel electrophoresis.

A correction has been made to the **MATERIALS AND METHODS section, in the sub-section Sample Preparation for nLC-MS/MS**:

Potential fungal spores were removed from the sap by centrifugation at 800 × *g* for 10 min. Xylem sap proteins were concentrated by passing 12 ml of cleared sap through Amicon Ultra-15 Filter Units (Millipore). After centrifugation at 2500 × *g* for 15–30 min retentates containing the proteins were recovered. A BCA (bicinchoninic acid) assay (ThermoFischer) was performed to determine the protein concentration. Based on BCA quantification, a volume containing 60 μg of protein was trichloroacetic acid/aceton-precipitated and the pellet was resuspended in SDS loading buffer (2% SDS, 10% glycerol, 60 mM TRIS-HCl pH 6.8, 5% β-mercaptoethanol, 0.01% bromophenol blue), heated at 98°C for 5 min and loaded on a 12% SDS-polyacrylamide gel. Following a short electrophoresis, the proteins were stained overnight at 4°C with Commassie PageBlue (ThermoFischer). The bands containing the proteins were excised and cysteine reduction and alkylation of the proteins was performed by adding 10 mM DTT pH 8 (incubation at 60°C for 1 h) and 20 mM iodoacetamide pH 8 (incubation at room temperature in the dark for 30 min). Protein-containing gel slices were chopped into pieces of approximately 1 mm^2^ and transferred to 1.5 ml low-binding tubes (Protein LoBind microcentrifuge tubes, Eppendorf). Tryptic in-gel digestion was performed overnight by adding 50 μl of 5 ng/μl Trypsin Sequencing Grade (Sigma-Aldrich). In-house prepared μcolumns were set up by adding C18 Empore disk and LichroprepC18 column material into a 200 μl pipette tip and the tryptic peptides were eluted from the μcolumn with 50 μl of 50% acetonitrile. Acetonitrile content was reduced to <5% by reducing the volume with a concentrator at 45°C during 2 h and readjusting the volume with 1 mL/L HCOOH in water to 50 μl.

The authors apologize for this error and state that this does not change the scientific conclusions of the article in any way. The original article has been updated.

