# Supplementary figures and images for: Xylem Sap Proteomics Reveals Distinct Differences Between R Gene- and Endophyte-Mediated Resistance Against Fusarium Wilt Disease in Tomato
Source: Front Microbiol. 2018 Dec 4;9:2977. doi: 10.3389/fmicb.2018.02977 (PMC6288350; doi:10.3389/fmicb.2018.02977)

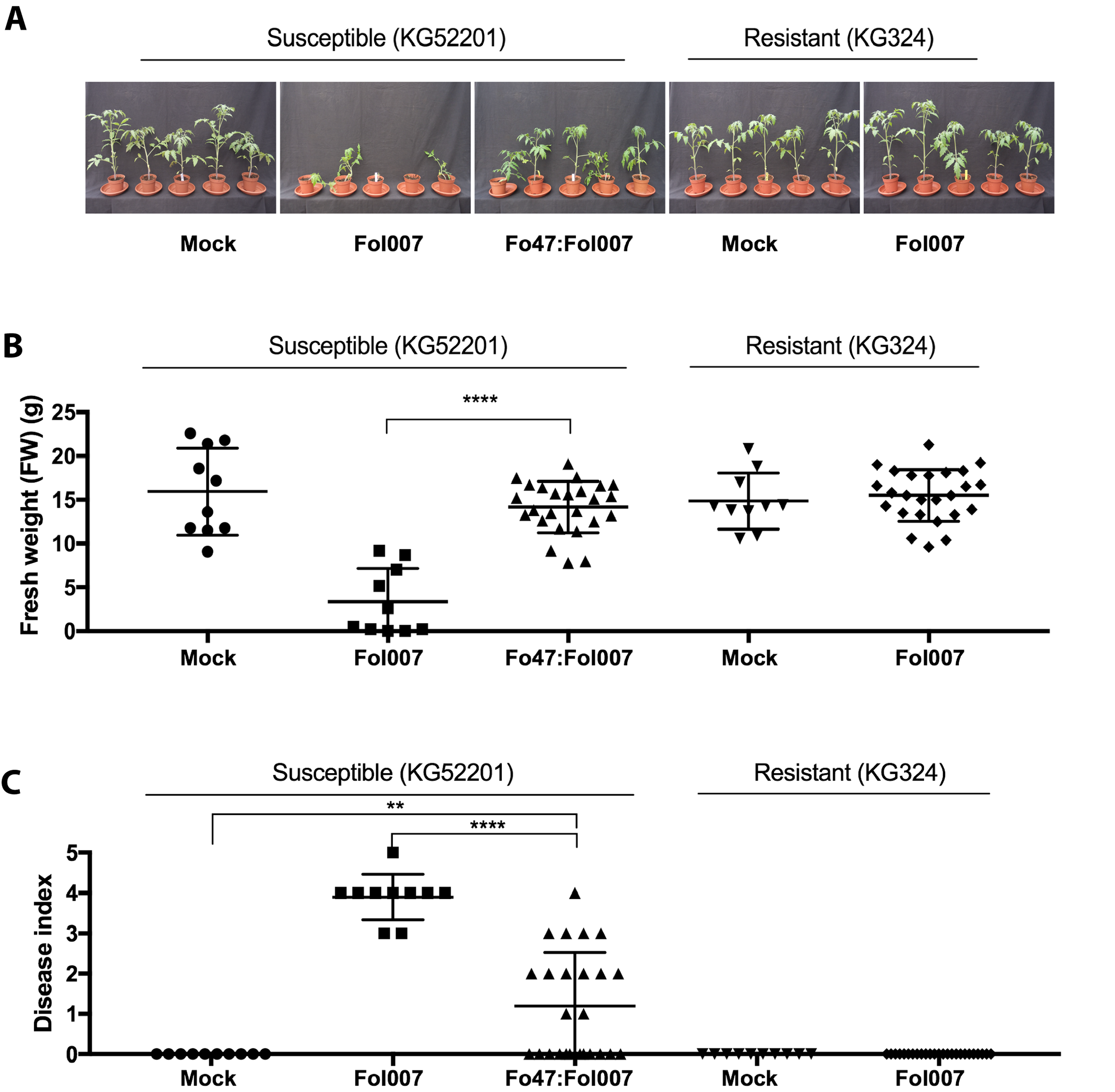

Supplement: FIGURE S1 — EMR and RMR reduce susceptibility to Fusarium wilt (Fol007). (A) Ten-days-old seedlings of Fol007-susceptible KG52201 and Fol007-resistant KG324 were root dip-inoculated with water (mock), Fol007 or a mixture of Fo47:Fol007 (25 replicates for Fo47:Fol007 co-inoculated KG52201 and Fol007-inoculated KG324 seedlings, 10 replicates for the control treatments). Three-weeks-post-inoculation (B) FW and (C) DI were scored (See Materials and Methods). The experiment was repeated three times yielding similar results (Figure 1 and Supplementary Figure S2). Error bars represent mean ±SD (∗∗Pval < 0.01, ∗∗∗Pval < 0.001, ∗∗∗∗Pval < 0.0001). An unpaired comparison for FW and DI was performed using the non-parametric Mann–Whitney U test. [file Image_1.TIF]

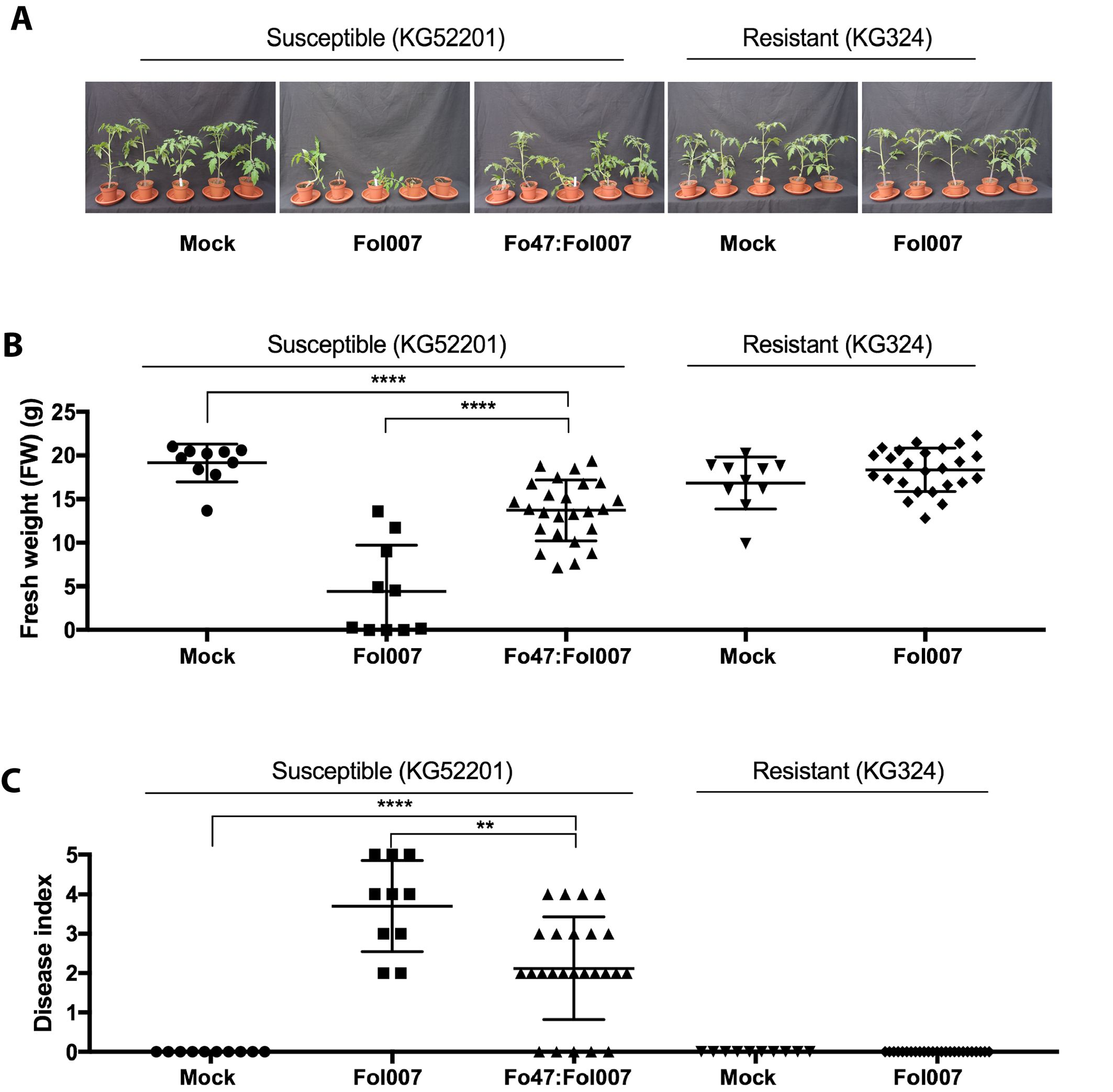

Supplement: FIGURE S2 — EMR and RMR reduce susceptibility to Fusarium wilt (Fol007). (A) Ten-days-old seedlings of Fol007-susceptible KG52201 and Fol007-resistant KG324 were root dip-inoculated with water (mock), Fol007 or a mixture of Fo47:Fol007 (25 replicates for Fo47:Fol007 co-inoculated KG52201 and Fol007-inoculated KG324 seedlings, 10 replicates for the control treatments). Three-weeks-post-inoculation (B) FW and (C) DI were scored (See Materials and Methods). The experiment was repeated three times yielding similar results (Figure 1 and Supplementary Figure S1). Error bars represent mean ±SD (∗∗Pval < 0.01, ∗∗∗Pval < 0.001, ∗∗∗∗Pval < 0.0001). An unpaired comparison for FW and DI was performed using the non-parametric Mann–Whitney U test. [file Image_2.TIF]

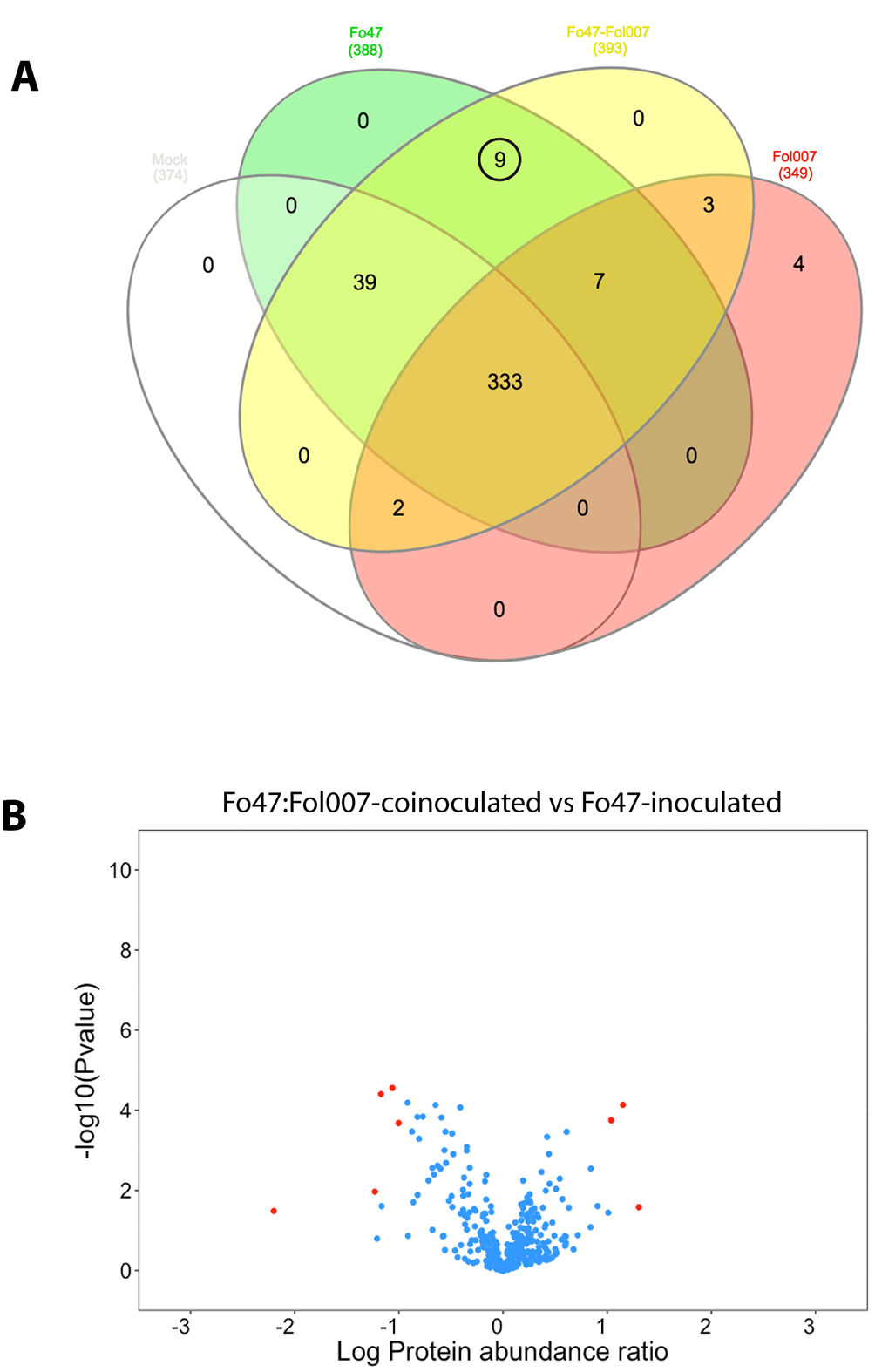

Supplement: FIGURE S3 — Fo47 inoculation affects accumulation of specific proteins. (A) Proteins that were detected in at least three out of four replicates where labeled as present. Treatments embraced mock-inoculated, Fo47-inoculated, Fo47:Fol007-coinoculated and Fol007-inoculated C32 susceptible tomato plants. The black circle marks proteins that were specifically present in Fo47 inoculated plants. (B) Volcano plot showing xylem sap protein abundance difference between Fo47:Fol007-coinoculated compared to Fo47-inoculated on susceptible C32 plants. Names of the DAPs are depicted in Supplementary Table S5. [file Image_3.TIF]

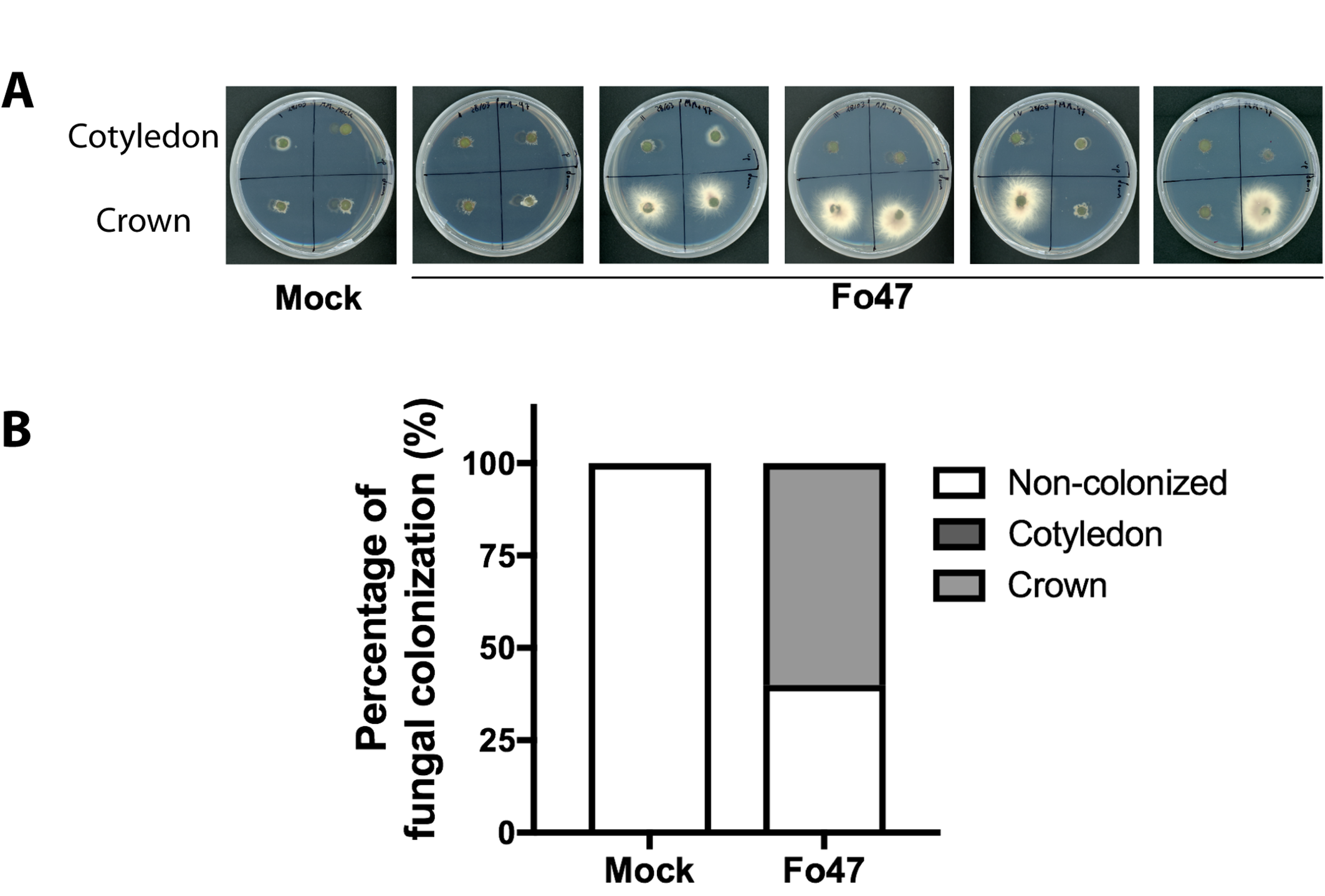

Supplement: FIGURE S4 — Fo47 colonizes tomato stems (A) To monitor stem colonization by Fo47 3-weeks-post-inoculation, stem sections at the crown and cotyledon-level were placed on PDA plates (10 replicates). Plates were scanned after 4 days of incubation. (B) Fungal outgrowth of the stem sections plotted as a percentage of infected sections. The experiment was repeated three times showing the same pattern. [file Image_4.TIF]
